# Selective Inhibition of Type III Secretion Activated Signaling by the *Salmonella* Effector AvrA

**DOI:** 10.1371/journal.ppat.1000595

**Published:** 2009-09-25

**Authors:** Fangyong Du, Jorge E. Galán

**Affiliations:** Section of Microbial Pathogenesis, Yale University School of Medicine, New Haven, Connecticut, United States of America; The Rockefeller University, United States of America

## Abstract

*Salmonella enterica* utilizes a type III secretion system (TTSS) encoded in its pathogenicity island 1 to mediate its initial interactions with intestinal epithelial cells, which are characterized by the stimulation of actin cytoskeleton reorganization and a profound reprogramming of gene expression. These responses result from the stimulation of Rho-family GTPases and downstream signaling pathways by specific effector proteins delivered by this TTSS. We show here that AvrA, an effector protein of this TTSS, specifically inhibits the *Salmonella-*induced activation of the JNK pathway through its interaction with MKK7, although it does not interfere with the bacterial infection-induced NF-κB activation. We also show that AvrA is phosphorylated at evolutionary conserved residues by a TTSS-effector-activated ERK pathway. This interplay between effector proteins delivered by the same TTSS highlights the remarkable complexity of these systems.

## Author Summary

*Salmonella* Typhimurium is a major cause of diarrheal disease worldwide. Central to *S.* Typhimurium's pathogenesis is its ability to induce intestinal inflammation, which is initiated by several bacterial proteins injected into intestinal epithelial cells by a nanomachine known as the type III secretion system. We show here that another bacterial protein injected by this machine negatively influences the responses triggered by *Salmonella*, presumably to limit cellular damage. This interplay between bacterial proteins of opposite function highlights the remarkable complexity of the host/pathogen interface.

## Introduction

Many bacteria that are pathogenic or symbiotic for plants, insects or animals utilize type III protein secretion systems (TTSSs) to modulate host-cellular functions through the activity of the effector proteins that they deliver [1],[2]. *Salmonella enterica* serovar Typhimurium (*S*. Typhimurium), an enteropathogen that causes inflammatory diarrhea, encodes two of such machines within its pathogenicity islands 1 (SPI-1) and 2 (SPI-2) [3] [4]. The SPI-1 TTSS is required for this pathogen to enter and replicate within intestinal epithelial cells [5]. In addition, this TTSS is required for the stimulation of innate immune responses in intestinal epithelial cells, which leads to inflammation and ultimately diarrhea [6],[7]. These responses are specifically triggered by the SPI-1 TTSS effector proteins, SopE, SopE2, and SopB, which activate Rho-family GTPases in a functionally redundant fashion [8],[9]. SopE and SopE2 are guanine nucleotide exchange factors (GEFs) for Rac, Cdc42, RhoA, and RhoG [10]–[12]. In contrast, SopB, through its phosphoinositide phosphatase activity, activates RhoG and Cdc42 by stimulating endogenous exchange factors for these GTPases [8]. Activation of these GTPases leads to the stimulation of NF-κB and the MAP kinases ERK, JNK, and p38 pathways, which ultimately result in a profound transcriptional reprogramming in epithelial cells closely resembling the responses that follow the stimulation of innate immune receptors [6],[9]. Therefore, the initiation of the stimulation of intestinal inflammation by this bacterium is dependent on specific bacterial adaptations rather than the hard-wired generic responses to a pathogen mediated by conserved innate immune receptors such as Toll-like (TLRs) or nucleotide oligomerization domain (NLRs) receptors [13],[14]. Indeed, the stimulation of inflammation is crucial for *Salmonella*'s ability to grow in the intestinal tract, since essential nutrients do not become available at this site unless such responses are stimulated [15].

Activation of Rho-family GTPases and its downstream MAP kinases or NF-κB signaling pathways can also result in significant alteration of the host cell homeostasis that may be detrimental for *Salmonella*'s ability to survive and replicate within these cells. Therefore, *Salmonella* has evolved a mechanism to down-regulate the responses triggered by its effector proteins. In a remarkable yin and yang, *S.* Typhimurium delivers through the SPI-1 TTSS an effector protein, SptP, which has GTPase-activating protein (GAP) activity directed towards a subset of the GTPases activated by SopE, SopE2 and SopB [16]. Thus, the activity of this bacterially-encoded GAP ensures the rapid reversion of the cytoskeletal changes induced by *Salmonella* infection.

AvrA is a SPI-1 TTSS effector protein that is a close homologue of YopJ, a *Yersinia* spp. TTSS effector protein that inhibits the activation of all MAP kinase kinases (MAP2Ks) and IκB kinases (IKKs) by acetylating critical residues for activation [17]–[19]. It has also been proposed that YopJ, and by extension AvrA, may have deubiquitinase or desumoylase activities [20]–[22]. However, these activities have been more recently brought into question and therefore remain the subject of some controversy [23],[24]. Despite their close amino acid sequence similarity, which suggests similar biochemical activity, AvrA does not phenocopy YopJ [25], indicating that these two effector proteins do not exert the same function and may target different cellular proteins. Although transient overexpression studies have identified some potential targets of AvrA [26],[27], its role during cell infection is still poorly understood. In this study, we present evidence that AvrA is another example of a SPI-1 TTSS effector, which like SptP [16], reverses the activation of specific signaling pathways induced by effectors delivered by *S.* Typhimurium *via* the same TTSS. We also report the identification of a naturally occurring allelic variant of AvrA with impaired activity, suggesting that this effector may be under strong evolutionary pressure to alter its function. In addition, we show that AvrA is phosphorylated upon translocation in an ERK-dependent manner, which may have implications for the regulation of the function of this effector protein.

## Results

### Expression of AvrA in *Saccharomyces cerevisiae* revealed an allelic variant with impaired function

Alignment of the annotated amino acid sequence of AvrA with that of its close homologue YopJ of *Yersinia* showed a 14 amino acid extension at the N-terminus of AvrA (Figure S1A). However, a second potential initiation codon located at amino acid 15 of the annotated open reading frame would generate an AvrA protein whose predicted amino acid sequence is more similar in length to YopJ. To ascertain which of these two putative initiation codons is functional, we changed either ATG codon and examined the expression of the resulting *avrA* mutants (Figure S1B). Mutation of the first predicted ATG initiation codon to GCC had little effect on the expression of AvrA in *S.* Typhimurium (Figure S1C). In contrast, mutation of the second putative initiation codon to GCC completely abolished *avrA* expression (Figure S1C). We concluded from these results that the second initiation codon is the correct translational start site and have therefore used for subsequent studies the coding sequence derived from this codon (Figure S1D), which differs from the originally annotated sequence.

**Figure 1:**
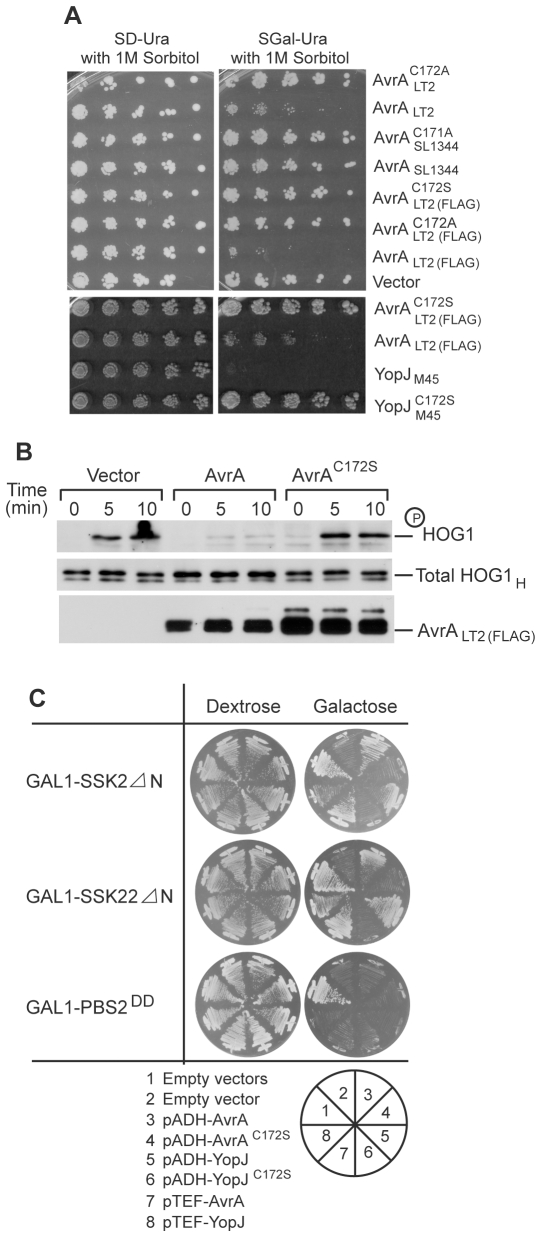
Expression of AvrA in *S. cerevisiae* inhibits the HOG MAP kinase pathway at the level of MAP2K PBS2. A Expression of the *avrA* allele from *S. typhimurium* LT2 strain, but not the allele from the *S. typhimurium* SL1344 strain, inhibits yeast growth in high osmolarity media. The yeast *Saccharomyces cerevisiae* strain NY605 (*MATa, leu2-3,112, ura3-52, GAL+)* carrying plasmids expressing the indicated alleles of *avrA* or *yopJ* were spotted onto selective dextrose or galactose plates containing 1 M sorbitol and the plates were then incubated at 30°C for 2 days. B Expression of AvrA inhibits the osmotically induced phosphorylation of the MAP kinase HOG1 of *S. cerevisiae* HOG pathway. Lysates from *S. cerevisiae* expressing either wild type AvrA or its catalytic mutant AvrA^C172S^ were analyzed by western immunoblot with antibodies directed to phosphorylated p38, and the HA and FLAG epitope tags (present in Hog1 and the AvrA constructs, respectively). C Expression of AvrA suppresses the lethality caused by constitutively active MAP3K mutants (SSK2ΔN and SSK22ΔN), but not by constitutively active MAP2K mutant (PBS2DD). *S. cerevisiae* strain NY605 expressing the indicated proteins were streaked onto plates containing either dextrose or galactose (to induce expression of the different *avrA* or *yopJ* constructs) and incubated at 30°C for 2 days.

It has been previously reported that in the yeast *S. cerevisiae,* the AvrA homologue YopJ inhibits multiple MAP kinase pathways, including the HOG pathway [28]. The HOG pathway in *S. cerevisiae* most closely resembles the p38 pathway in mammals. We tested whether AvrA would inhibit the HOG pathway when expressed in the yeast *S. cerevisiae* by examining whether AvrA inhibits yeast growth in high osmolarity conditions, since adaptation to growth in high osmolarity media is dependent on the HOG pathway [29]. In contrast to YopJ, expression of AvrA in *S. cerevisiae* did not prevent growth in high osmolarity media (Figure 1A). This result was surprising given the high degree of amino acid sequence similarity between YopJ and AvrA. We therefore examined the sequence of the *avrA* allele from the *S.* Typhimurium strain SL1344 used in this experiment and found that, in comparison to alleles from other strains of *S.* Typhimurium such as LT2 [30], the SL1344 *avrA* allele has a three nucleotide deletion resulting in the absence of a leucine residue at position 139 (Leu139) (Figure S2A). This change is not due to a sequencing error since independently sequenced copies of *avrA* from SL1344 deposited in GenBank exhibit the same variation. Furthermore, we have re-sequenced *avrA* from *S.* Typhimurium SL1344 and confirmed the presence of this allelic variation. Interestingly, this allele is unique to SL1344 since all other sequenced alleles of AvrA in the Genbank encode Leu139 (Figure S2B and data not shown). Leu139 is located in a conserved region of this protein family raising the possibility that this mutation may be functionally significant. To investigate this hypothesis, we expressed in yeast an *avrA* allele in which the three nucleotides (TTT) were inserted into the SL1344-derived *avrA* after nucleotide 413 of the coding sequence, thus creating an *avrA* allele identical to the one encoded in the *S.* Typhimurium LT2 strain (AvrA_LT2_). Expression of *avrA*_LT2_ inhibited yeast growth under high osmolarity conditions (Figure 1A), and this inhibitory activity was strictly dependent on the catalytic activity of AvrA since a catalytic mutant derivative AvrA_LT2_^C172A^ did not inhibit yeast growth (Figure 1A). Furthermore, similar to YopJ [28], this growth inhibition was due to suppression of the HOG pathway by AvrA_LT2_ , since overexpression of AvrA_LT2_ inhibited phosphorylation of HOG1 without altering its total protein level after high osmolarity stress (Figure 1B). More specifically, overexpressed AvrA_LT2_ exerted its effect at the level of the MAP2K PBS2, since it was able to suppress the lethal effects caused by overexpression of constitutively active MAP kinase kinase kinases (MAP3Ks) SSK2 or SSK22 mutants, but not by overexpression of the constitutively active PBS2 mutant (Figure 1C). Taken together, these results indicate that, like YopJ, AvrA_LT2_ is capable of inhibiting MAP kinase signaling in yeast and that the *S.* Typhimurium SL1344 strain harbors an allele incapable of exerting this activity. All subsequent studies involving AvrA reported here were carried out with the LT2 allele of AvrA (or mutant derivatives as indicated).

### Transiently expressed or TTSS-delivered AvrA is phosphorylated in mammalian cells

**Figure 2:**
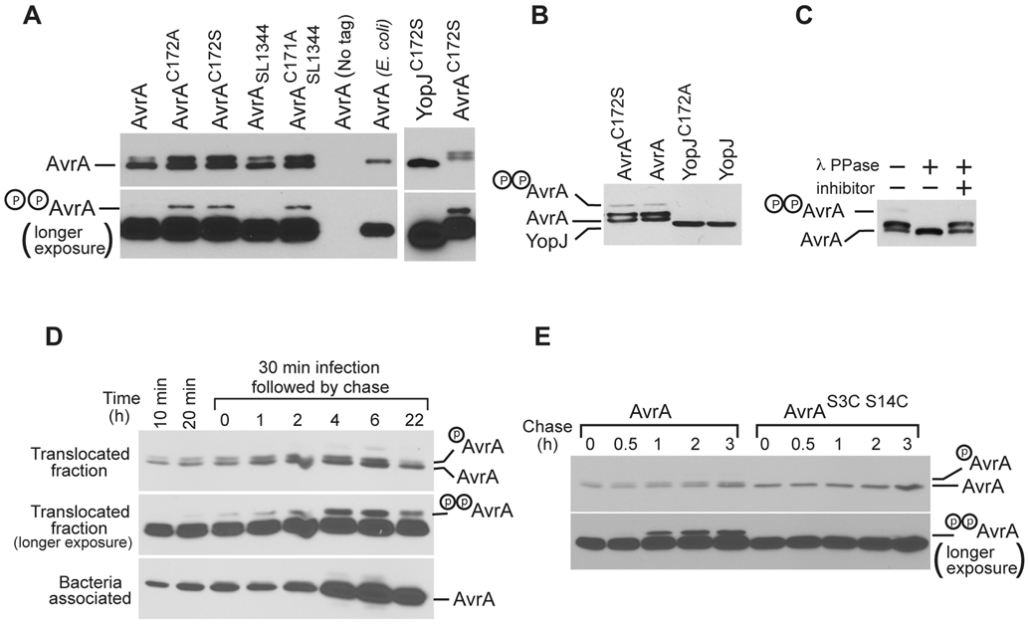
AvrA is phosphorylated when expressed in *S. cerevisiae* or in mammalian cells. A AvrA is phosphorylated in *S. cerevisiae*. Extracts of *S. cerevisiae* expressing the indicated FLAG epitope tagged alleles of AvrA or YopJ were analyzed by western immunoblot with antibodies directed to the FLAG epitope. The lower panel shows longer exposure of the gel to visualize the dually phosphorylated form of AvrA (see text for details). B Transiently expressed AvrA is phosphorylated in mammalian cells. COS cells were transfected with plasmid expressing FLAG epitope tagged AvrA, YopJ, or their catalytic mutants AvrA^C172S^ and YopJ^C172S^. Twenty-four hours after transfection, cell extracts were examined by western immunoblot with an antibody directed to the FLAG epitope. C The mobility shift of transiently expressed AvrA is reversed after phosphatase treatment. COS cells were transfected with plasmid expressing FLAG epitope tagged AvrA and twenty-four hours after transfection, cell extracts were treated with lambda phosphatase in the presence or absence of a phosphatase inhibitor and examined by western immunoblot as indicated above. D AvrA is phosphorylated and persists within mammalian cells after its delivery via the *S.* Typhimurium TTSS. Henle-407 cells were infected with a *S.* Typhimurium strain expressing FLAG epitope-tagged AvrA and at the indicated times after infection, the presence of AvrA in the translocated and bacteria-associated protein fractions was probed by western immunoblot with an anti FLAG antibody. The lower panels show a longer exposure of the gel to visualize the dually phosphorylated form of AvrA. E Ser3 and Ser14 are essential for AvrA phosphorylation in mammalian cells. Henle-407 cells were infected with a *S.* Typhimurium strain expressing FLAG epitope-tagged AvrA or its mutant AvrA^S3C S14C^ and at the indicated times after infection, the presence of AvrA in the translocated and bacteria-associated protein fractions was probed by western immunoblot with an anti FLAG antibody. The lower panels show a longer exposure of the gel to visualize the dually phosphorylated form of AvrA.

We noticed that when AvrA was expressed in *S.* Typhimurium or *E.coli*, it migrates as a single band (Figure S1C). However, when expressed in yeast, AvrA migrates as a triplet (Figures 1B and 2A). We found that this mobility shift could be reversed by treatment with the non-specific λ phosphatase (data not shown), suggesting that it is due to its phosphorylation (data not shown). In contrast, when expressed in yeast, YopJ migrated as a single band (Figure 2A) indicating that it is not phosphorylated. Since AvrA and YopJ differ significantly in their N-terminal 20 residues (Figure S1D), we hypothesized that the sequences responsible for AvrA phosphorylation were located within these amino acids. Indeed, removal of the N-terminal 20 residues of AvrA abolished its mobility shift (Figure S3A). There are several serine or threonine residues within this region that could serve as potential phosphorylation sites (Figure S3B), and mutagenesis analysis indicated that Ser3 and Ser14 are essential for AvrA phosphorylation in yeast (Figure S3C).

We tested whether AvrA could be phosphorylated in mammalian cells, a more physiologically relevant model for *S.* Typhimurium infection. Similar to what we observed in yeast, when transiently expressed in mammalian cells and separated by SDS-PAGE, AvrA, but not YopJ, migrated as a triplet (Figure 2B). Treatment of the samples with the non-specific λ phosphatase eliminated the mobility shift (Figure 2C), demonstrating that phosphorylation of AvrA was responsible for its mobility shift. Like in yeast, mutations of Ser3 and Ser14 abolished the mobility shift indicating that these residues are critical for phosphorylation (Figure S3D).

We then tested whether AvrA was phosphorylated after its delivery by the *S.* Typhimurium SPI-1 TTSS. Cultured intestinal epithelial cells were infected with a strain of *S.* Typhimurium expressing FLAG epitope tagged AvrA_LT2_ or its derivatives carrying mutations in Ser3 and Ser14 from its native promoter in a low-copy plasmid, and the presence of AvrA in the translocated protein fraction was analyzed over time. AvrA was translocated into mammalian cells shortly after infection, and remained detectable for several hours after infection (Figure 2D). Furthermore, translocated AvrA exhibited a migration pattern in SDS-PAGE consistent with its phosphorylation at both Ser3 and Ser14 (Figure 2E). The mobility in SDS-PAGE of the translocated epitope tagged AvrA^Ser3^ or AvrA^Ser14^ mutants confirmed that, like in yeast, these two residues are essential for phosphorylation when this effector is delivered during infection (Figure S3E). Interestingly, both Ser3 and Ser14 are followed by a proline residue, and these residues are highly conserved among AvrA alleles of different *Salmonella enterica* serovars (Figure S2B), including the most distant *Salmonella enterica* serovar S. Bongori (Figure S3F). Taken together, these results indicate that shortly after delivery by the type III secretion, AvrA is rapidly phosphorylated at two sites, and that phosphorylated AvrA remains within the cell for an extended period of time after infection.

### The ERK pathway, which is activated upon *S*. Typhimurium infection, is required for the phosphorylation of AvrA in cultured intestinal epithelial cells

**Figure 3:**
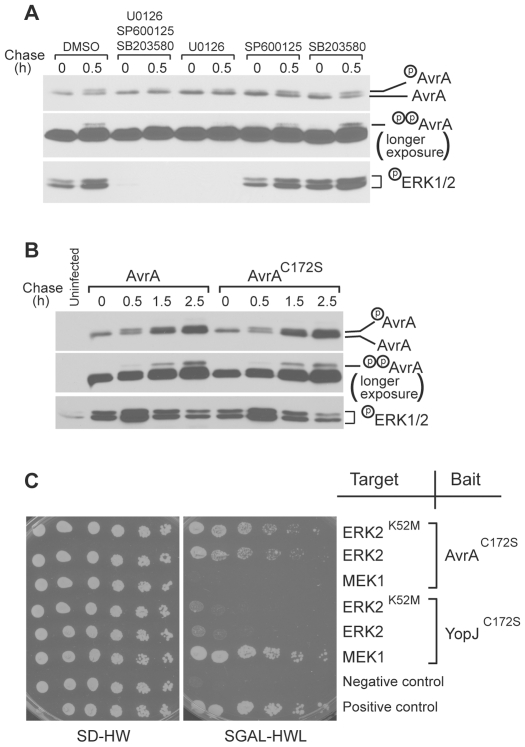
The ERK signaling pathway is required for AvrA phosphorylation. A Effect of inhibitiors of MAP kinase pathways on AvrA phosphorylation. Cultured Henle cells were incubated with 10 µM each (alone or in combination) of the ERK (UO126), JNK (SP600125), or p38 (SB203580) MAP kinase pathways 30 min prior to *S. typhimurium* infection and were left throughout the experiment. Cells were infected with a *S.* Typhimurium strain expressing C-terminally triple FLAG tagged AvrA and at the indicated times after infection, the presence of AvrA and activated ERK1/2 in the translocated protein fractions were probed by western immunoblot with an anti FLAG or anti phospho-p44/42 MAPK antibodies respectively. The middle panel shows a longer exposure of the gel to visualize the dually phosphorylated form of AvrA. B *S.* Typhimurium *ΔavrA* strain expressing wild type AvrA or its catalytic mutant AvrA^C172S^ activates ERK to similar levels. Henle-407 cells infected with the indicated *S.* Typhimurium strains for the indicated times and cell lysates were probed by western immunoblot with an anti FLAG or anti phospho-p44/42 MAPK antibodies respectively. The middle panel shows a longer exposure of the gel to visualize the dually phosphorylated form of AvrA. C AvrA interacts with ERK2 but not MEK1 while YopJ interacts with MEK1 but not ERK2 in a yeast two-hybrid assay. AvrA^C172S^ or YopJ^C172S^ were expressed in yeast as baits along with the ERK pathway components as the targets as indicated in the diagram. See Materials and Methods for details.

The observation that both putative phosphorylation sites Ser3 and Ser14 are followed by a proline residue suggests that a proline-directed kinase(s) is responsible for their phosphorylation. Since MAP kinases are the major group of proline-directed kinases, we specifically tested whether AvrA is phosphorylated by MAP kinases. To identify the MAP kinases involved in AvrA phosphorylation in mammalian cells, we investigated the effect of specific MAP kinase inhibitors on the SDS-PAGE migration pattern of translocated AvrA after bacterial infection. Treatment of cells with JNK (SP600125) or p38 (SB203580) specific inhibitors had no measurable effect on the migration pattern of translocated AvrA (Figure 3A), indicating that these kinases are not involved in the phosphorylation of AvrA. In contrast, translocated AvrA in cells treated with a specific MEK1/2 inhibitor (UO126) migrated largely as a single (unmodified) band, implicating the ERK pathway in AvrA phosphorylation (Figure 3A). Addition of any of these inhibitors had no effect on the levels of translocated AvrA after infection (Figure 3A).

The observation that the ERK pathway was required for AvrA phosphorylation was surprising since YopJ, a close homologue of AvrA, was shown to inhibit all three MAP kinase pathways, including the ERK pathway [31]. We therefore tested whether AvrA was also able to inhibit the ERK pathway. As shown previously [6], infection of mammalian cells with *S.* Typhimurium resulted in rapid activation of the ERK pathway (Figure 3B). However, infection with a strain expressing wild type or a catalytic mutant of AvrA resulted in similar levels of ERK activation and AvrA phosphorylation (Figure 3B). These results indicate that, unlike YopJ, AvrA does not inhibit the ERK pathway when translocated through the type III secretion system. This conclusion is consistent with the finding that while YopJ interacted with MEK1 in a yeast two-hybrid assay, AvrA did not (Figure 3C). Instead, AvrA, but not YopJ, interacted with ERK2 in a yeast two-hybrid assay (Figure 3C), suggesting that AvrA might be a direct substrate of the proline-directed ERK1/2 kinase itself. These results indicate that when delivered through the type III secretion system, AvrA is phosphorylated by the Salmonella-activated ERK pathway, and targets different signaling pathways than those targeted by its homologue YopJ.

### Delivery of AvrA through the SPI-1 type III secretion system inhibits *Salmonella*-induced JNK pathway but not the NF-κB pathway

**Figure 4:**
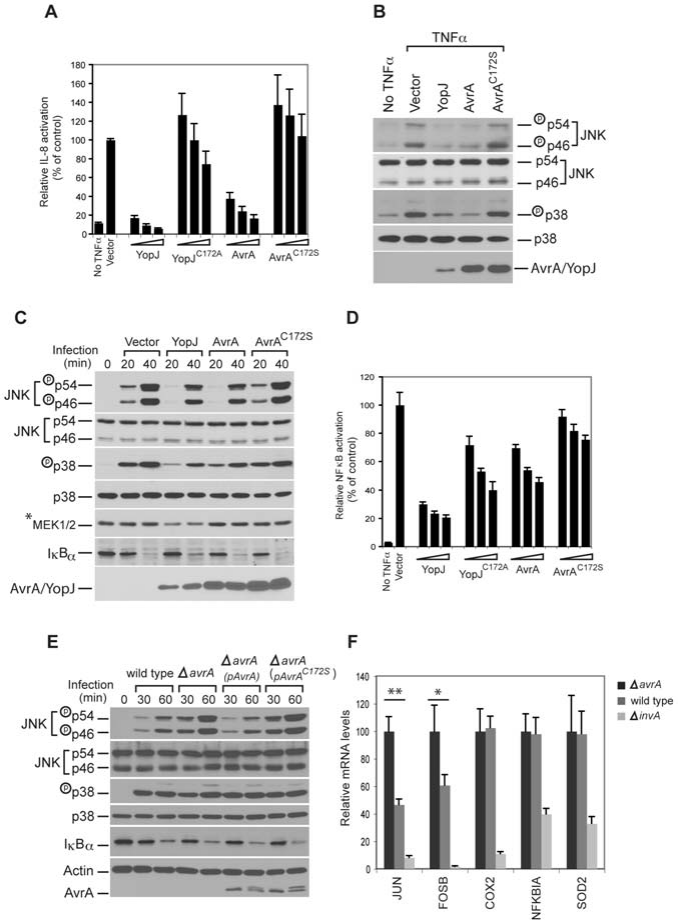
AvrA delivered by the TTSS inhibits the activation of the JNK but not the p38 or NF-κB pathways stimulated by *S. typhimurium* infection. A Transiently overexpressed AvrA inhibits TNFα-stimulated IL-8 transcription. HEK293 cells were co-transfected with a pIL8-luc firefly luciferase reporter plasmid along with a plasmid encoding renilla luciferase (to standardize transfection), and when indicated, with a plasmid encoding AvrA, YopJ or their catalytic mutants AvrA^C172S^ or YopJ^C172A^. Twenty-four hours after transfection, cells were treated with TNFα (10 ng/ml) for 30 min and the stimulation of IL-8 transcription in transfected cells was measured with the Dual-Luciferase Reporter Assay System (Promega) as indicated in Materials and Methods. Values represent fold induction in cells transfected with the plasmid vector alone and are the mean±standard deviation of three independent measurements. B and C Transiently overexpressed AvrA inhibits TNFα- and *Salmonella* infection-stimulated JNK and p38 pathways. HEK293 cells were transfected with the indicated plasmids and 24 hs after transfection, cells were either treated with TNFα (10 ng/ml) or infected with *S.* Typhimurium *ΔavrA* strain. Thirty minutes after treatment or at the indicated times after infection the activation of p38 and JNK was assayed by western immunoblot using antibodies directed to the phosphorylated (activated) form of these kinases (see Materials and Methods for details). D Transiently overexpressed AvrA inhibits TNFα-stimulated NF-κB promoter reporter transcription only very weakly. The assay was performed in the same way as in A, except that the reporter plasmid pBIIX-Luc was used, which harbors two tandem repeats of NF-κB recognition sites. Values represent fold induction in cells transfected with the plasmid vector alone and are the mean±standard deviation of three independent measurements. E AvrA delivered by the *S.* Typhimurium TTSS inhibits the JNK signaling pathway but does not affect the p38 or NF-κB pathways. HEK293 cells were infected with *S.* Typhimurium strains expressing AvrA or its catalytic mutant AvrA^C172S^ and at the indicated times after infection, the stimulation of p38 and JNK, as well as stabilization of IκBα, were assayed by western immunoblot as described in Materials and Methods. F Quantitative RT-PCR analysis of selected genes induced by *S. typhimurium* infection. Henle-407 intestinal epithelial cells were infected for 1 h with *S. typhimurium* wild type, *ΔavrA* or the type III secretion-defective *ΔinvA* strains, and then chased in DMEM supplemented with gentamicin (100 µg/ml) for 3 hs. RT-qPCR was performed as described in Materials and Methods. COX2, NFKBIA, and SOD2 are regulated by the NF-κB pathway, while JUN and FOSB are regulated by the JNK pathway. The threshold cycles for the specified genes were normalized against the reference gene GAPDH. Values represent mRNA fold induction relative to that of *ΔinvA* strains and are the mean±standard deviation of four independent measurements. ** = *P*<0.001; * = *P*<0.05 (student *t* test, relative to wild type values).

Previous studies have shown that, like YopJ, transient overexpression of AvrA inhibited the NF-κB and JNK pathways [26],[27]. We were able to confirm that overexpression of AvrA in cultured mammalian cells resulted in the inhibition of TNFα-induced IL-8 promoter reporter stimulation, which is dependent NF-κB (Figure 4A), as well as TNFα-stimulated JNK and p38 phosphorylation (Figure 4B). We further demonstrated that overexpression of AvrA inhibited the JNK and p38 pathways activated by *S.* Typhimurium infection (Figure 4C). However, overexpression of AvrA only slightly stabilized IκBα after Salmonella infection (Figure 4C), suggesting that overexpressed AvrA may only inhibit the NF-κB pathway very weakly. Consistent with this conclusion, overexpression of AvrA resulted in a very weak inhibition of the NF-κB promoter reporter after addition of TNFα (Figure 4D).

Transient transfection experiments, however, lead to expression levels of effector proteins that are usually orders of magnitude higher than those achieved during bacterial infection. Non-physiological levels of expression of an effector protein could artificially alter its specificity and hence confound the interpretation of the results. Furthermore, during infection, AvrA operates in conjunction with other effector proteins that have the capacity to activate the specific pathways that could also be targeted by AvrA itself. Consequently, the study of AvrA function without the bacterial infection context could lead to misleading results. We therefore tested the function of AvrA in the context of bacterial infection of cultured mammalian cells. We found that contrary to its effect in transient transfection experiments, AvrA had no effect on the *Salmonella* infection-induced activation of the p38 or NF-κB signaling pathways (Figure 4E). We observed the same level of activation of these two signaling pathways whether cells were infected with *S.* Typhimurium expressing active AvrA or an isogenic *ΔavrA* mutant (Figure 4E). In contrast, JNK phosphorylation (a measure of its activation) was significantly inhibited in cells infected with *S.* Typhimurium expressing active AvrA when compared to cells infected with an isogenic *ΔavrA* mutant (Figure 4E). This inhibition was specifically dependent on AvrA since it could be complemented by introducing into the *ΔavrA* mutant a plasmid encoding AvrA but not its catalytic mutant (Figure 4E). These results indicate that AvrA translocated through TTSS modulates the degree of activation of the *Salmonella* infection-induced JNK signaling pathways.

Since AvrA translocated through TTSS inhibits *Salmonella* infection-induced JNK pathway, but not the NF-κB pathway, we further investigated whether AvrA modulated the qualitative and/or quantitative output of the SPI-1-induced transcriptional reprogramming stimulated by *S.* Typhimurium in cultured intestinal epithelial cells. We infected cultured intestinal epithelial cells with wild type, the isogenic *ΔavrA* mutant, or the isogenic type III secretion-defective *ΔinvA* mutant, and compared their transcriptional response profiles using DNA microarray analysis. The list of genes that were upregulated after *Salmonella* infection was very similar to what was found in previous microarray analyses (Table S1) [9], indicating a high degree of reproducibility of this assay. Interestingly, the induced expression levels of most genes, many of which are known to be regulated by the NF-κB pathway, were not affected by AvrA (Table S1). The expression of three representative genes COX2, NFKBIA, and SOD2, which are known to be regulated by NF-κB, were further analyzed using quantitative RT-PCR (Figure 4F). No differences were detected between their level of induction after infection with wild type or the *ΔavrA* mutant strains. These results further support the conclusion that AvrA translocated through TTSS does not affect *Salmonella* infection-induced NF-κB pathway. However, the expression of a small group of genes was stimulated to significantly higher levels in cells infected with the *S.* Typhimurium *ΔavrA* mutant than with the wild type (Table S1), suggesting that TTSS-delivered AvrA inhibited their induction. Using quantitative RT-PCR we confirmed this observation for JUN and FOSB, two of the genes in this group. Interestingly, expression of JUN and FOSB has been reported to be strongly dependent on the transcription factor AP1, which in turn is activated by the JNK pathway [32]. Therefore the higher expression of these genes in cells infected with the *ΔavrA* mutant is consistent with the observation that AvrA inhibits the JNK pathway. Taken together, these results indicate that AvrA can specifically influence the transcriptional output stimulated by effectors of its SPI-1 TTSS during *Salmonella* infection. In this context, the function of AvrA is conceptually analogous to that of SptP, which also inhibits pathways initially activated by *Salmonella* SPI-1 TTSS effectors. These two effectors, AvrA and SptP, may even cooperate with each other since both are capable of inhibiting *Salmonella* infection-induced JNK activation albeit by different mechanisms [16]. These results also underscore the pitfalls of transient overexpression experiments since, in the case of AvrA, these experiments assigned some phenotype to AvrA that was not observed when it was delivered by *Salmonella* TTSS.

### AvrA specifically interacts with MKK7

**Figure 5:**
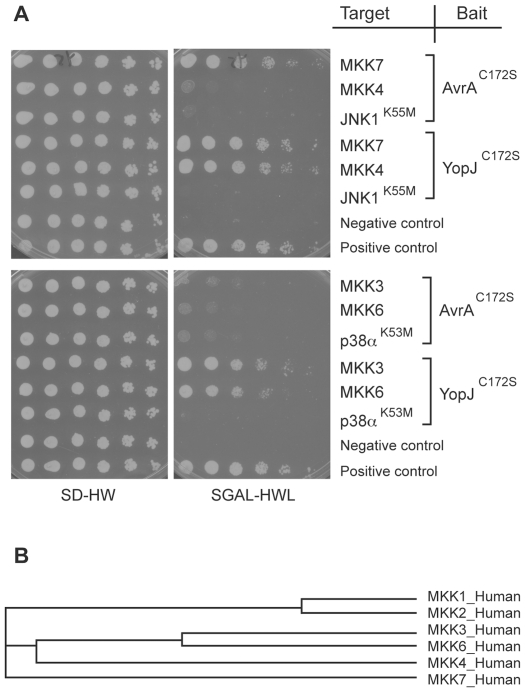
AvrA specifically interacts with MKK7 in a yeast two-hybrid assay. A AvrA^C172S^ or YopJ^C172S^ was expressed in yeast as a bait along with the JNK or p38 pathway components as the targets as indicated in the diagram. See Materials and Methods for details. B Phylogenetic analysis of the human MKKs. The cladogram was generated using ClustalW2 [42].

YopJ, the close homologue of AvrA, inhibits MAP kinase and NF-κB pathways by specifically interacting with MKKs and IKKs, and acetylating critical Ser and Thr residues [17]–[19]. Stimulation of two MAP2Ks, MKK4 or MKK7, leads to JNK activation. Howewer, MKK4 and MKK7 are not redundant since they are activated by different stimuli and have different substrate specifity: MKK7 is specific to the JNK pathway, while MKK4 can also activate p38 pathway [33]. Transient overexpression of AvrA was shown to inhibit MKK4 and MKK7-mediated JNK phosphorylation, and to acetylate MKK4 at Lys260 and Thr261 [26],[27]. However, the effect of AvrA over these kinases when delivered during bacterial infection by the SPI-TTSS, or the interactions between MKK4, MKK7 and AvrA have not been reported. We performed yeast two-hybrid assays with AvrA^C172S^ or YopJ^C172S^ as baits, and components of the JNK and p38 pathways as targets. We confirmed that YopJ^C172S^ interacted with all the MAP2Ks tested (MKK3, MKK6, MKK4 and MKK7) (Figure 5A). However, AvrA^C172S^ only interacted with MKK7 (Figure 5A), the only MAP2K that is specific to the JNK pathway. Phylogenetic analysis revealed that MKK7 is most distant from the rest of MAP2Ks (Figure 5B), and therefore may exhibit the most unique structural features to be specifically targeted by AvrA. The specific interaction between AvrA amd MKK7 is consistent with the observed specific inhibition of the JNK pathway by AvrA when delivered at physiological concentrations. Furthermore these results indicate that AvrA, unlike YopJ, has evolved to narrow its substrate to only one MAP2K.

### Effect of phosphorylation on AvrA function

**Figure 6:**
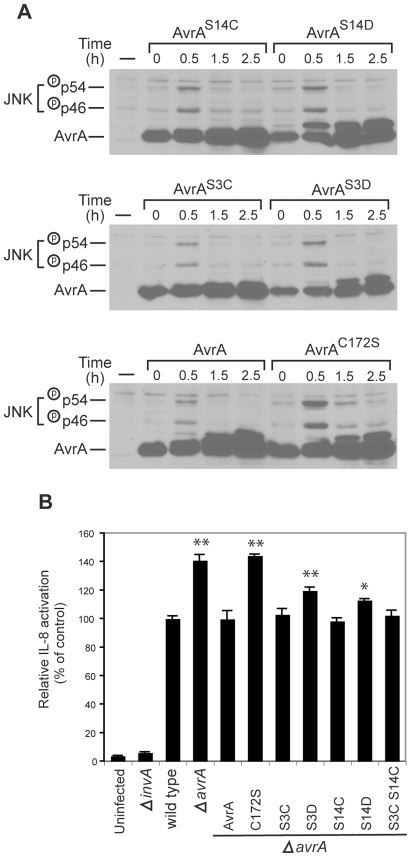
Effect of phosphorylation on AvrA function. A Effect of phosphorylation on the ability of AvrA to inhibt *S.* Typhimurium-activated JNK activation. Henle-407 cells were infected for 30 min with a *ΔavrA* strain harboring a low copy plasmid expressing FLAG-epitope tagged AvrA or the indicated mutants (all expressed from the *avrA* native promoter), then chased in DMEM supplemented with gentamicin (100 µg/ml) for the indicated times. The activation of JNK and the phosphorylation of AvrA were monitored by western immunoblot analysis as indicated above. B Effect of AvrA phosphorylaton on its ability to inhibit *S.* Typhimurium-stimulated IL-8 transcription. HEK293 cells were co-transfected with pIL8-luc firefly luciferase reporter plasmid along with a plasmid encoding renilla luciferase (to standardize transfection). Twenty four hours after transfection, cells were infected with *S.* Typhimurium strains expressing the indicated AvrA mutants and the stimulation of IL-8 transcription in infected cells was measured with the Dual-Luciferase Reporter Assay System (Promega) as indicated in Materials and Methods. Values represent fold induction in cells transfected with the plasmid vector alone and are the mean±standard deviation of three independent measurements. ** = *P*<0.001; * = *P*<0.05 (student *t* test, relative to wild type values).

We next investigated the potential influence of AvrA phosphorylation on its function when delivered by the *S.* Typhimurium SPI-1 TTSS. Cultured intestinal epithelial cells were infected with *S.* Typhimurium strains expressing the phosphorylation site mutants AvrA^S3C^, AvrA^S3D^, AvrA^S14D^, or AvrA^S14C^ from the AvrA native promoter in a low-copy plasmid, and the levels of effector protein translocation and JNK activation after infection were evaluated by western immunoblot analysis over time. Cells infected with *S.* Typhimurium expressing AvrA^S14D^ showed increased levels of JNK activation in comparison to cells infected with a strain expressing wild type AvrA or AvrA^S14C^, despite similar levels of protein translocation (Figure 6A). AvrA^S14D^ carries a mutation that mimics the change in charge that results from its phosphorylation at Ser14. These results indicate that introduction of a phospho mimic mutation reduces AvrA activity and therefore suggest that phosphorylation may have a negative effect on AvrA function. Cells infected with *S.* Typhimurium expressing AvrA^S3D^ also showed increased level of JNK phosphorylation relative to cells infected with s strain expressing AvrA^S3C^ or wild type (Figure 6A). However, this was probably due to reduced levels of AvrA^S3D^ translocation (Figures 6A). In no case did the phosphorylation mutants phenocopy the catalytic mutant AvrA^C172S^ (Figure 6A).

We also measured the effect of the phosphorylation site mutants using a more quantitative IL-8 luciferase transcription reporter system. In this assay, cells infected with *S.* Typhimurium expressing wild type AvrA exhibited a reproducibly lower (∼50%) levels of IL-8 transcription reporter activity when compared with cells infected with an isogenic *ΔavrA* strain (Figure 6B). While cells infected with strains expressing AvrA^S3C^ or AvrA^S14C^ show similar levels of IL-8 transcription reporter activity as those infected with wild type, cells infected with strains expressing the phosphomimic site mutants AvrA^S3D^ or AvrA^S14D^ exhibited a slight but reproducibly higher level of IL-8 transcription reporter activity (Figure 6B). Taken together, these results suggest that phosphorylation may have a negative effect on AvrA activity.

## Discussion

*Salmonella* Typhimurium has evolved a very complex functional interface with its host, the product of evolutionary forces operating over an extended time of coexistence. At the center of this functional interface are two TTSSs, which work in concert to modulate different aspects of *Salmonella/*host interactions [3],[4]. One of these systems, encoded within the SPI-1, mediates the initial interaction of *S.* Typhimurium with intestinal epithelial cells. Although the function of only a subset of the proteins delivered by this system is known, it is already evident that this system mediates at least two very important aspects of *Salmonella*/host interactions: 1) entry and survival within intestinal epithelial cells; and 2) stimulation of a profound transcriptional reprogramming in intestinal epithelial cells that sets in motion an inflammatory response. Both of these activities, which are dependent on the ability of *S.* Typhimurium to activate members of the Rho-family GTPases and the resulting activation of MAP kinase and NF-κB signaling pathways, are crucial for *Salmonella*'s ability to replicate within the host and spread to other hosts. In particular, the stimulation of an inflammatory response is essential for *Salmonella*'s ability to acquire scarce nutrients [15] and, through the stimulation of diarrhea, to spread in the environment. However, the stimulation of these responses does have potentially negative effects. For example, potent stimulation of Rho-family GTPase members may lead to programmed cell death [34], which may result in the premature demise of cells actually “hosting” *Salmonella* and serving as a “safe place” for their replication and survival. Furthermore, a very strong inflammatory response may have negative consequences on *Salmonella*'s long-term survival. *Salmonella* has evolved mechanisms to limit these potentially harmful responses. For example, *Salmonella* has evolved the ability to specifically counter the activation of Rho-family GTPases. In a remarkable yin and yang, *Salmonella* counters the function of the SPI-1 effector proteins that activate Rho-family GTPases by delivering through the same TTSS an effector protein with GAP activity towards the GTPases activated by those effectors [16]. In this study, we have presented evidence of another example of a SPI-1 effector protein that counters the functional consequence of the activities of other SPI-1 effector proteins. Indeed, we have shown here that AvrA, through its catalytic activity, specifically counters the SPI-1-TTSS effector protein activated JNK pathway, thus modulating the transcriptional reprogramming that results from this activation.

Previous studies using transient overexpression of AvrA have reported that AvrA can also inhibit NF-κB signaling pathways [26]. We have shown here that overexpression of AvrA inhibts NF-κB signaling pathway only very weakly, but we have obtained no evidence that *S.* Typhimurium inhibits NF-κB activation in intestinal epithelial cells during bacterial infection. This is in keeping with the profound pro-inflammatory nature of this bacterium, and underscores the intrinsic difficulties of attempting to glean the specific activity of an effector protein using overexpression studies. Indeed, transient overexpression often leads to protein levels orders of magnitude higher than the usually very low levels reached by effector proteins after their delivery through TTSSs. These much higher levels of a given effector protein can lead to a loss of specificity and hence artifactual effects on cellular physiology.

Our results are also in keeping with a previous report indicating that *S.* Typhimurium can inhibit JNK pathway [27] and highlight the significant differences between AvrA and its *Yersinia* spp. homologue YopJ. While AvrA when delivered in physiological amount has a rather narrow substrate specificity, inhibiting only the JNK pathway, YopJ seems to have a much broader spectrum of targets, inhibiting essentially all MAP kinase pathways as well as NF-κB pathway [23],[31]. Consistent with functional assays, yeast two-hybrid assays revealed that AvrA only interacts with MKK7 while YopJ interacts with all the MAP2Ks tested. Therefore it seems that evolution has tailored the activity of these highly related proteins to suit the actual physiology of the bacteria that harbor them. In the case of *Yersinia* spp., an extracellular pathogen, YopJ has evolved to broadly inhibit the host innate immune response. In contrast, the function of AvrA is more subtle and not aimed at blocking the innate immune responses of intestinal cells but rather at modulating them. This is consistent with the need for *Salmonella* to induce inflammation to secure nutrients and spread to other hosts [15]. The apparently rather narrow spectrum of targets for AvrA activity which is directed to only the JNK pathway, is also in keeping with the fact that AvrA itself is a target of the ERK pathway, a MAP kinase pathway not targeted by this effector protein. Although the role of phosphorylation on AvrA function is still unclear, it is remarkable that the phosphorylation sites are highly conserved across *Salmonella* serovars including the most distantly related *Salmonella* Bongori. It is also intriguing that the activity of AvrA may be modulated by a signaling pathway that is stimulated by *Salmonella* infection.

Although clearly measurable, the phenotype of the *ΔavrA* mutant is subtle in our *in vitro* assays. This is consistent with the observation that in a mouse model of infection, absence of AvrA results in no difference in the number of colony forming units recovered from different tissues at different times after infection when compared with wild type (unpublished observations). This may mean that AvrA function is not critical in this specific animal model of infection and/or that the *in vitro* assays used in this (and other) studies are not adequate to probe its function. It is indeed possible that AvrA may also target other signaling pathways in addition to the JNK pathway. A deeper understanding of *Salmonella* pathogenesis may be required to glean other potential functions of AvrA.

We have reported here that the *S.* Typhimurium strain SL1344 encodes an AvrA allele with different functional properties from those encoded by other *S.* Typhimurium strains. Although this allele is apparently not active against the targets tested in this study, it remains possible that it retains activity against other, yet to be identified, targets. In this context, it is interesting that some *Salmonella enterica* serovars such as *S.* Typhi or *S.* Choleraesuis do not encode *avrA* [17], and we have found that in some strains of *S.* Typhimurium there are mutations within the *avrA* coding sequence that render the protein non-functional in the assays utilized in these studies (unpublished observations). It therefore appears that this effector protein may be under selection pressure to alter its specificity or, in some cases, to even completely lose its function.

In summary, we have shown here that AvrA specifically modulates the output of signaling pathways triggered by *S.* Typhimurium through the activity of SPI-1 effector proteins. Along with SptP, AvrA is another example of an effector protein whose activity is exerted in the context of the activities of other effector proteins delivered by the same TTSS system. The coordinated activity of TTSS effector proteins highlights the remarkable complexity of these systems, which have been evolved presumably to operate on both the pathogens and their hosts.

## Materials and Methods

### Plasmid constructs

The plasmids used in yeast growth assay were derived from p416GAL1 [35]. The Open Reading Frames (ORFs) of AvrA, YopJ or their variants were inserted between the Hind III and Xho I sites downstream of the GAL1 promoter. Mutagenesis of plasmids were performed with a QuikChange Site-Directed Mutagenesis kit (Stratagene, CA). The plasmids pGSS21, pGYC731 and pGPBD21 expressing GAL1-SSK2ΔN, GAL1-SSK22ΔN and GAL1-PBS2(DD) respectively were kindly provided by Dr. Tatsuya Maeda (University of Tokyo, Tokyo, Japan). The plasmid pIL-8-Luc has been described [8]. The AvrA expressing plasmids used in mammalian cell transfections were constructed by subcloning the ORFs encoding AvrA or their derivatives into the *Hind*III and *Xho*I sites of pCDNA3.1 (Invitrogen). The YopJ expressing plasmids used in mammalian cell transfections were constructed by subcloning the ORFs encoding YopJ or their derivatives into the *BamH I* and *Xba* I sites of the vector pRK5Flag. The plasmids used to express proteins in *S.* Typhimurium were derived from pWKS130 [36]. The DNA fragments encoding C-terminally 3xFLAG tagged AvrA or its mutants as well as the native AvrA promoter were cloned into the Sac I and Kpn I sites of pWKS130. The details of plasmid construction are available upon request.

### Bacterial strains

All strains were derived from the *S. enterica* serovar Typhimurium (*S.* Typhimurium) strain SL1344 [37]. The Δ*avrA* (SB1117) [17] and Δ*invA* (SB136) [38] mutants have been described previously. The *S.* Typhimurium strains SB1432 and SB1434 were constructed by allelic exchange as previously described [39]. SB1432 encodes an *avrA* containing three additional nucleotides (TTT) coding for a leucine at position 139 of the amino acid sequence, which correspondes to the *S.* Typhimurium LT2 allele of *avrA*. This strain was used as wild type in all these studies. SB1434 is a derivative of SB1432 encoding a C-terminal 3xFLAG tagged version of the modified AvrA. All bacterial strains were cultured under conditions that stimulate the expression of the *Salmonella* pathogenicity island-1–encoded TTSS [40]. Briefly, overnight cultures were diluted 1∶25 in L-broth containing 0.3 M NaCl, incubated on a rotating wheel for 3 h at 37°C until an optical density measured at 600 nanometers (OD_600_) of 0.9. The cultures were then used immediately for infection.

### Cell culture and bacterial infection

Henle-407 intestinal epithelial cells were routinely grown in 6-well plates in DMEM supplemented with 10% bovine calf serum (BCS; HyClone). Fifteen hours before infection, cells were washed once with DMEM and incubated in DMEM overnight. Ten minutes before infection, cells were washed once with pre-warmed HBSS and incubated in 1 ml of HBSS at 37°C. Unless specified, cells were infected with the indicated *S.* Typhimurium strains at an MOI 30 for 1 h, and subsequently incubated in DMEM supplemented with gentamicin (100 µg/ml) to kill extracellular bacteria. Cells were then harvested at the indicated times and samples were processed for Western blotting.

### Yeast handling and growth assay

The yeast *Saccharomyces cerevisiae* strain NY605 (*MATa, leu2-3,112, ura3-52, GAL+)* was transformed with various plasmids according to standard protocol [41]. The transformants were grown in selective yeast media containing 0.67% yeast nitrogen base without amino acids, and proper auxotrophic amino acids at the standard concentrations [41]. The carbon sources were either 2% glucose or 2% galactose, as indicated. Yeast transformants were grown to an OD_600_∼0.6, 1 ml of cells were then spun down and resuspended into 1 ml of water. The samples were serially diluted by 4-fold in a 96-well plate. The suspensions were then spotted to plates containing selective SD or SGal media with 1 M sorbitol using a 48-pin applicator. The plates were then incubated at 30°C for 2 days. For experiments described in Figure 1C, the yeast *S. cerevisiae* strain NY605 was transformed with two types of plasmids. One type of plasmids expressed the constitutively active MAP3K (SSK2ΔN and SSK22ΔN) or MAP2K (PBS2^DD^), from the galactose-inducible pGAL1 promoter. The other type of plasmids expressed from a constitutive promoter pADH1 (or pTEF) AvrA, YopJ, or their active site mutants AvrA^C172S^ or YopJ^C172S^. The transformants were re-streaked onto either dextrose or galactose selective plates and incubated at 30°C for 2 days.

### Extraction of proteins from yeast cells and immunoblotting

The yeast transformants were grown in 2 mls of selective media to an OD_600_ of 1.0. Cultures were mixed with equal volumes of cold 40% TCA, spun down and resuspended into 300 µl of 1× SDS-PAGE sample buffer containing 100 mM DTT in a screw-cap tube. Glass beads (∼250 µl) were added to each tube and yeast cells were broken open with mini-bead beater for 3 times, 1 min each time. The yeast extracts were then heated at 60°C for 5 min and cooled on ice. The debris were removed by centrifugation at 14,000 rpm for 5 min, the pHs of the supernatants were neutralized with 1 M Tris base, and then were separated by SDS-PAGE. When indicated, the yeast transformants were also grown in selective liquid media containing the non-inducing sugar raffinose until OD_600_∼0.8, galactose was then added to a final concentration of 2%, and 3 h later, 2 M NaCl stock solution was added to a final concentration of 0.4 M. Five and 10 min after this treatment, samples were taken and processed for immunoblotting as described above.

### Mammalian cell transfection and protein extraction

COS-2 and HEK293 cells were maintained in DMEM (Invitrogen) supplemented with 10% heat-inactivated FCS (Gemini). Plasmids were transfected into mammalian cells using FuGENE 6 Transfection Reagents (Roche, IN) at the ratio of 3∶1 (3 ul of FuGENE6 reagent for 1 µg of plasmid). Usually 1 µg of total plasmid was used for each well of a 6-well plate. Immediately before harvesting, cultured cells were rinsed with cold HBSS, lysed in Extraction Buffer A (20 mM Tris-HCl [pH 7.5], 150 mM NaCl, 25 mM β-glycerophosphate, 0.5% NP40, 0.5 mM DTT, 1 mM PMSF and 1 mM sodium orthovanadate), and soluble cellular lysates analyzed by western immunoblotting.

### Bacteriophage lambda phosphatase treatment of AvrA

COS-2 cells transfected with an AvrA-expressing plasmid were lysed in lysis buffer (25 mM Tris-HCl [pH 7.6], 50 mM NaCl, 0.1% Triton X-100, 1 mM PMSF) at 4°C, cell lysates (80 µg of protein) were incubated with 400 U of lambda phosphatase (New England Biolabs, MA ) at 30°C for 40 min, and the reaction was then terminated by addition of SDS-containing loading buffer. When indicated, 10 mM sodium orthovanadate was included as an inhibitor of the lambda phosphatase.

### Luciferase reporter assay

HEK293 cells were transfected with the reporter plasmid pIL8-Luc, which encodes the firefly luciferase gene under the control of IL8 promoter, along with a control plasmid (pRL-TK) encoding renilla luciferase, and plasmids encoding either YopJ or AvrA. 24 h after transfection, cells were washed once with fresh DMEM, and stimulated with TNFα (10 ng/ml) for 30 min in DMEM. Cells were further incubated in fresh DMEM for another 5 h before lysis in Cell Culture Lysis Reagent (Promega). Luciferase activity was measured with the Dual-Luciferase Reporter Assay System (Promega) using a luminometer following the recommendations of the manufacturer All experiments were done in triplicates. In some experiments, HEK293 cells were transfected with the reporter plasmid pIL8-Luc and the control plasmid pRL-TK, and 24 hs after transfection, cells were washed once with fresh HBSS, and infected with different strains of *S.* Typhimurium with a MOI of 10 for 30 min. Cells were then washed once with pre-warmed DMEM, and incubated in DMEM supplemented with gentamicin (100 g/ml) for another 5 hs to kill extracellular bacteria. Cells were then lysed in Cell Culture Lysis Reagent (Promega), and luciferase activities were measured as described above. All experiments were done in triplicates.

### Yeast two-hybrid analysis

The yeast two-hybrid analysis was performed using the DupLEX-A Yeast Two-Hybrid System (OriGene Technologies) following the instructions of the manufacturer. The DNA fragments encoding AvrA^C172S^ or YopJ^C172S^ were cloned between the EcoR I and Not I sites of the bait vector pEG202, and the DNA fragments encoding MEK1, ERK2, ERK2^K52M^, MKK3, MKK6, p38α^K53M^, MKK4, MKK7, and JNK1^K55M^ were cloned between the *Sma*I and *Xho*I sites of the target vector pJG4-5. Both the bait and target plasmids were co-transformed into the yeast strain EGY48 and transformants were plated onto SD-HIS-TRP. Yeast transformants were grown in selective media to an OD_600_∼0.6, 1 ml of cells were then spun down and its OD_600_ was adjusted to 1.0 with water. The samples were serially diluted by 4-fold in a 96-well plate. The suspensions were then spotted onto SD-HIS-TRP-LEU and SGAL-HIS-TRP-LEU plates. The SD-HIS-TRP-LEU plates were incubated at 30°C for 2 days while the SGAL-HIS-TRP-LEU plates were incubated at 30°C for 4 days.

### Microarray gene expression profiling

Total RNA was isolated from the infected Henle-407 cells using TRIzol reagent (Invitrogen) following the manufacturer's protocol, followed by on-column digestion of DNA using the RNeasy Mini Kit (Qiagen). Samples were proceessed and hybridized to Human Gene 1.0 ST Array (Affymetrix) at the Yale University W. M. Keck facility. The data were analysed with NetAffx. Fold change in gene expression were calculated for each *S. typhimurium* strain relative to the type III secretion-defective *S.* Typhimurium Δ*invA* mutant strain.

### Quantitative real-time PCR

Total RNA was isolated from infected Henle-407 cells using TRIzol reagent (Invitrogen) following the manufacturer's protocol, followed by on-column digestion of DNA using the RNeasy Mini Kit (Qiagen). Reverse transcription of RNA was performed using iScript cDNA Synthesis Kit (Bio-Rad) following the manufacturer's instructions. The cDNA mix was subject to RT-qPCR using iQ SYBR Green Supermix (Bio-Rad). Reactions were run in an iCycler real time PCR machine (Bio-Rad) and data were obtained and analyzed using the iCycler iQ System (Bio-Rad). The threshold cycles for the specified genes were normalized against the reference gene GAPDH. RNA concentrations were measured using Nanodrop ND-1000 Spectrophotometer (Nanodrop). The gene-specific oligonucleotide pairs used for the qRT-PCR reactions are as follows:

GAPDH: 5′-GTCCACTGGCGTCTTCAC-3′; 5′-CTTGAGGCTGTTGTCATACTTC-3′

JUN 5′: TGCCTCCAAGTGCCGAAAAA-3′; 5′-TGACTTTCTGTTTAAGCTGTGCC-3′

FOSB 5′-TTTCCCCGGAGACTACGACTC-3′; 5′-CTGGTTGTGATCGCGGTGA-3′

COX2 5′: ATATGTTCTCCTGCCTACTGGAA-3′; 5′GCCCTTCACGTTATTGCAGATG-3′.

NF-κB-IA: 5′-CCGCACCTCCACTCCATCC-3′; 5′-ACATCAGCACCCAAGGACACC-3′

SOD2 5′: CTGCTGGGGATTGATGTGTGG-3′; 5′-TGCAAGCCATGTATCTTTCAGT-3′

IL-8 5′: AGAGACAGCAGAGCACACAAG-3′; 5′-AATCAGGAAGGCTGCCAAGAG-3′

## Supporting Information

Figure S1 (A) Nucleotide sequence surrounding the two putative initiation codons of *avrA* as compared to that of *yopJ*. The putative Shine-Dalgarno sequence of *avrA* is boxed. (B) A diagram showing mutations of the two putative initiation codons (ATG) of *avrA* to GCC. (C) AvrA is not expressed when the second putative initiation codon is replaced by GCC. C-terminally M45 epitope tagged AvrA or the mutants were expressed from its native promoter in a low-copy plasmid in either *S. typhimurium* wild type or a type III secretion-defective *ΔinvG* strains. The lysate was probed by western immunoblot with an anti M45 antibody. (D) Amino acid sequence alignment of AvrA_LT2_ and YopJ. The active sites are denoted with an arrow and the residue Leu139 that is missing in *S. typhimurium* SL1344 strain is boxed.
(4.59 MB TIF)

Figure S2 (A) Alignment of nucleotide sequences from *avrA* and *yopJ* surrounding the three nucleotides (TTT) that are missing in *S. typhimurium* SL1344 strain. (B) Variations of amino acid sequence of AvrA from several *Salmonella enterica* serovars. Leu139 is missing only in the *S. typhimurium* SL1344 strain.
(2.03 MB TIF)

Figure S3 Identification of Ser3 and Ser14 of AvrA as the phosphorylation sites in yeast and mammalian cells. (A) AvrA lacking its first 20 amino acids is not phosphorylated in *S. cerevisiae*. Extracts of *S. cerevisiae* expressing the indicated FLAG epitope tagged alleles of AvrA were analyzed by western immunoblot with antibodies directed to the FLAG epitope. The lower panel shows longer exposure of the gel. (B) Amino acid sequence alignment of the N-terminal 20 residues of AvrA and YopJ. Identical residues are boxed and the two stretches of Ser/Thr residues of AvrA are underlined. (C) Ser3 and Ser14 are essential for AvrA phosphorylation in yeast. Extracts of *S. cerevisiae* expressing the indicated FLAG epitope tagged alleles of AvrA were analyzed by western immunoblot with antibodies directed to the FLAG epitope. The lower panel shows longer exposure of the gel to visualize the dually phosphorylated form of AvrA. (D) Ser3 and Ser14 are essential for AvrA phosphorylation in mammalian cells after transfection. COS cells were transfected with plasmid expressing the indicated FLAG epitope tagged AvrA mutants and twenty four hours after transfection, cell extracts were analyzed by western immunoblot as indicated above. The lower panel shows a longer exposure of the gel to visualize the dually phosphorylated form of AvrA. (E) Ser3 and Ser14 are essential for AvrA phosphorylation in mammalian cells after delivery by the TTSS. Henle-407 cells were infected with a *S*. Typhimurium strain expressing FLAG epitope-tagged AvrA or its mutant AvrA^S3C^, AvrA^S3D^, AvrA^S14C^, or AvrA^S14D^. At the indicated times after infection, the presence of AvrA in the translocated protein fractions was probed by western immunoblot with an anti FLAG antibody. The lower panels show a longer exposure of the gel to visualize the dually phosphorylated form of AvrA. (F) Amino acid sequence alignment of the N-terminal 20 residues of AvrA_LT2_ and AvrA_Bongori_. The two phosphorylation sites Ser3 and Ser14 as well as their following Pro residues are conserved. The identical residues are boxed.
(3.67 MB TIF)

Table S1 Microarray analysis of the transcriptional responses induced by different strains of *Salmonella* Typhimurium.
(0.05 MB PDF)
