# Supplementary figures and images for: Selective Inhibition of Type III Secretion Activated Signaling by the Salmonella Effector AvrA
Source: PLoS Pathog. 2009 Sep 25;5(9):e1000595. doi: 10.1371/journal.ppat.1000595 (PMC2742890; doi:10.1371/journal.ppat.1000595)

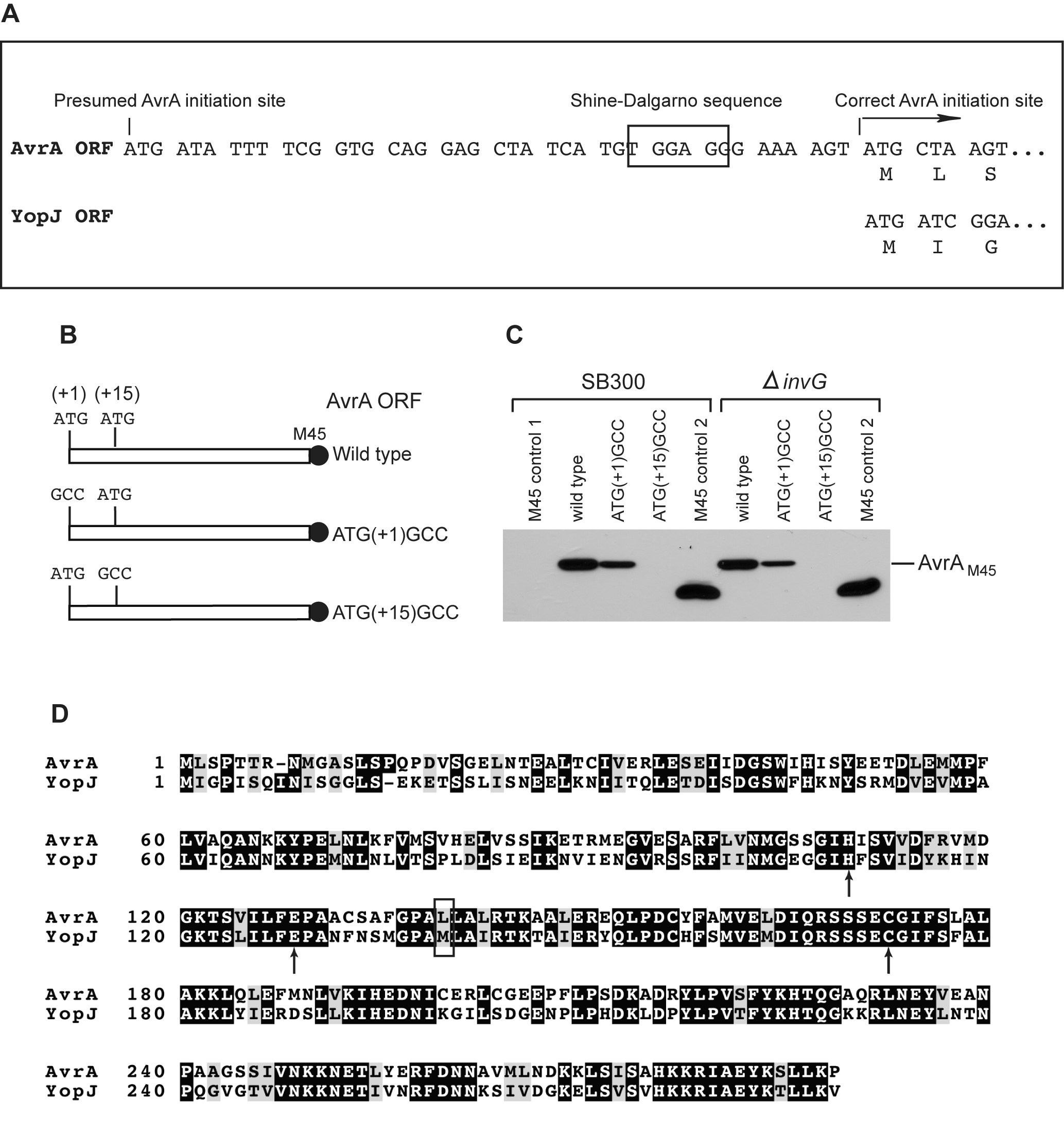

Supplement: Figure S1 — (A) Nucleotide sequence surrounding the two putative initiation codons of avrA as compared to that of yopJ. The putative Shine-Dalgarno sequence of avrA is boxed. (B) A diagram showing mutations of the two putative initiation codons (ATG) of avrA to GCC. (C) AvrA is not expressed when the second putative initiation codon is replaced by GCC. C-terminally M45 epitope tagged AvrA or the mutants were expressed from its native promoter in a low-copy plasmid in either S. typhimurium wild type or a type III secretion-defective ΔinvG strains. The lysate was probed by western immunoblot with an anti M45 antibody. (D) Amino acid sequence alignment of AvrALT2 and YopJ. The active sites are denoted with an arrow and the residue Leu139 that is missing in S. typhimurium SL1344 strain is boxed. (4.59 MB TIF) [file ppat.1000595.s001.tif]

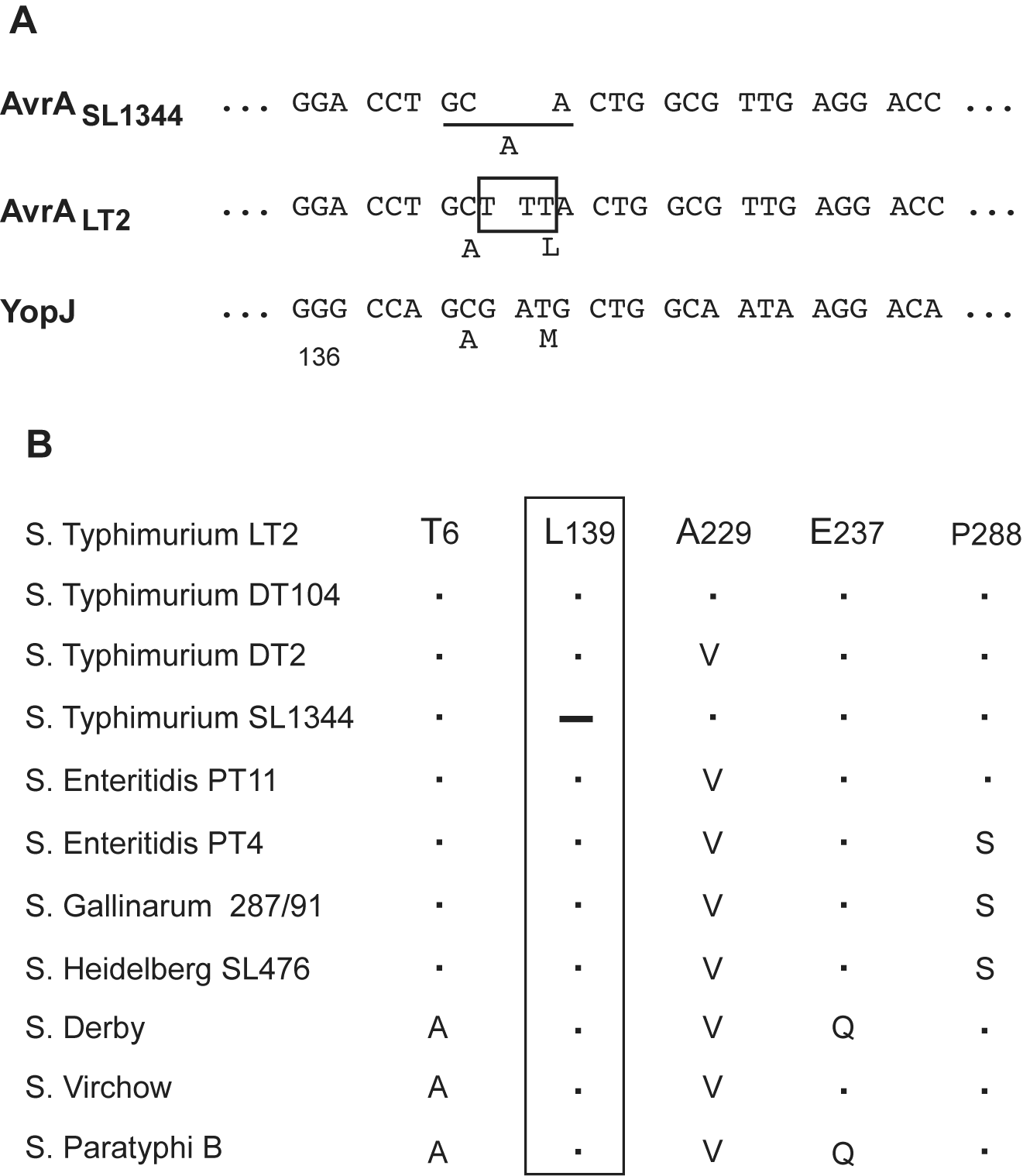

Supplement: Figure S2 — (A) Alignment of nucleotide sequences from avrA and yopJ surrounding the three nucleotides (TTT) that are missing in S. typhimurium SL1344 strain. (B) Variations of amino acid sequence of AvrA from several Salmonella enterica serovars. Leu139 is missing only in the S. typhimurium SL1344 strain. (2.03 MB TIF) [file ppat.1000595.s002.tif]

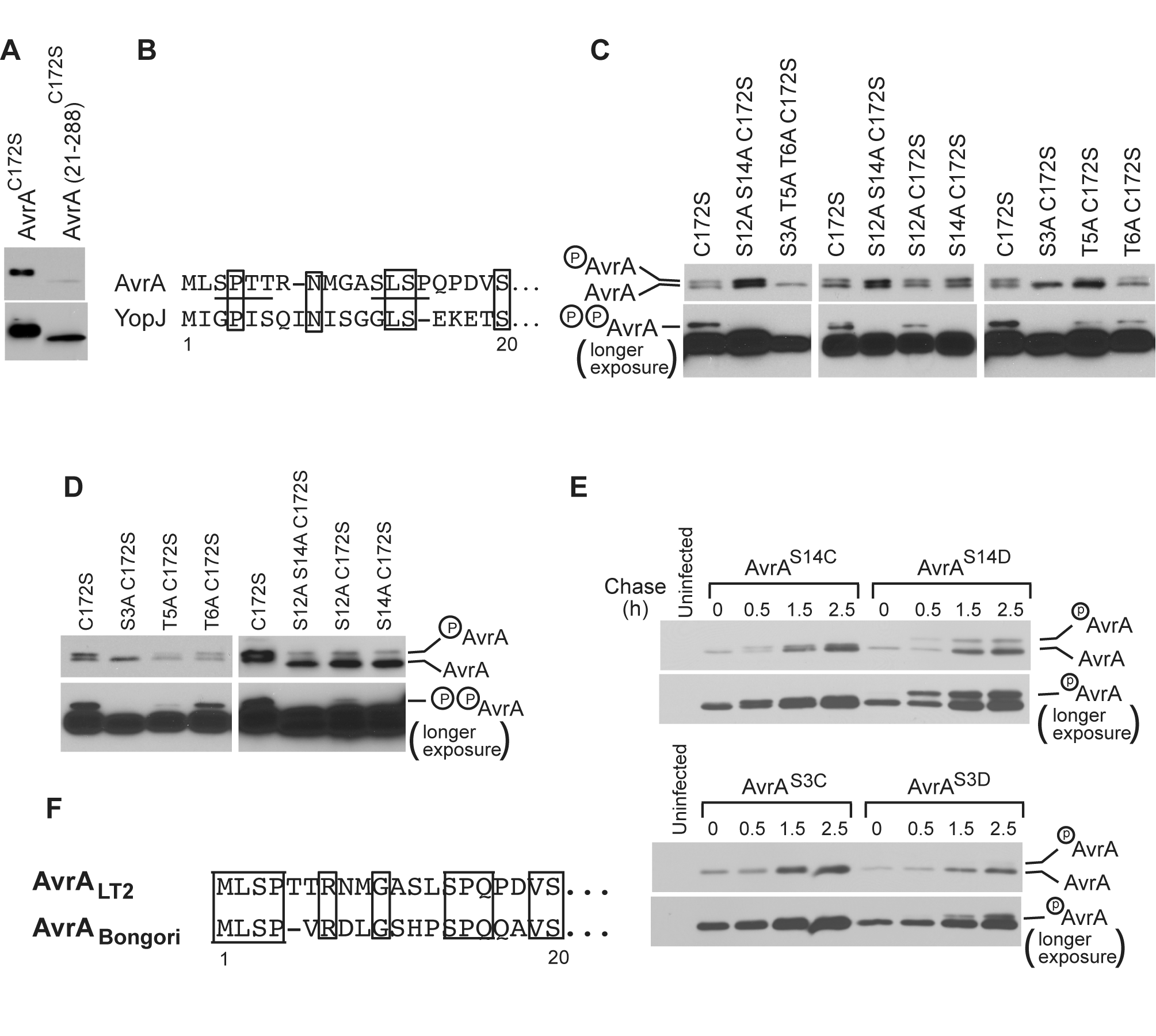

Supplement: Figure S3 — Identification of Ser3 and Ser14 of AvrA as the phosphorylation sites in yeast and mammalian cells. (A) AvrA lacking its first 20 amino acids is not phosphorylated in S. cerevisiae. Extracts of S. cerevisiae expressing the indicated FLAG epitope tagged alleles of AvrA were analyzed by western immunoblot with antibodies directed to the FLAG epitope. The lower panel shows longer exposure of the gel. (B) Amino acid sequence alignment of the N-terminal 20 residues of AvrA and YopJ. Identical residues are boxed and the two stretches of Ser/Thr residues of AvrA are underlined. (C) Ser3 and Ser14 are essential for AvrA phosphorylation in yeast. Extracts of S. cerevisiae expressing the indicated FLAG epitope tagged alleles of AvrA were analyzed by western immunoblot with antibodies directed to the FLAG epitope. The lower panel shows longer exposure of the gel to visualize the dually phosphorylated form of AvrA. (D) Ser3 and Ser14 are essential for AvrA phosphorylation in mammalian cells after transfection. COS cells were transfected with plasmid expressing the indicated FLAG epitope tagged AvrA mutants and twenty four hours after transfection, cell extracts were analyzed by western immunoblot as indicated above. The lower panel shows a longer exposure of the gel to visualize the dually phosphorylated form of AvrA. (E) Ser3 and Ser14 are essential for AvrA phosphorylation in mammalian cells after delivery by the TTSS. Henle-407 cells were infected with a S. Typhimurium strain expressing FLAG epitope-tagged AvrA or its mutant AvrAS3C, AvrAS3D, AvrAS14C, or AvrAS14D. At the indicated times after infection, the presence of AvrA in the translocated protein fractions was probed by western immunoblot with an anti FLAG antibody. The lower panels show a longer exposure of the gel to visualize the dually phosphorylated form of AvrA. (F) Amino acid sequence alignment of the N-terminal 20 residues of AvrALT2 and AvrABongori. The two phosphorylation sites Ser3 and Ser14 as well as their [file ppat.1000595.s003.tif]
