# Supplementary material for: Selective Inhibition of Type III Secretion Activated Signaling by the Salmonella Effector AvrA
Source: PLoS Pathog. 2009 Sep 25;5(9):e1000595. doi: 10.1371/journal.ppat.1000595 (PMC2742890; doi:10.1371/journal.ppat.1000595)
Supplement: Table S1 — Microarray analysis of the transcriptional responses induced by different strains of Salmonella Typhimurium. (0.05 MB PDF) [file ppat.1000595.s004.pdf]

Table S1: Microarray analysis of the transcriptional responses induced by dif
strains of *Salmonella Typhimurium*

Probe Set	Gene	wild type	Δ*avrA*	WT/Δ*avrA*
7959102	HSPB8	1.7	3.1	0.55
8112198	ACTBL2	1.7	2.9	0.57
7916609	JUN	3.5	5.6	0.62
7931810	KLF6	2.0	2.8	0.70
7943969	HTR3A	1.7	2.3	0.72
7909610	ATF3	1.6	2.2	0.73
8144526	LOC100129150	1.9	2.6	0.74
8115831	DUSP1	2.5	3.4	0.74
8084794	IL1RAP	2.2	3.0	0.75
8029693	FOSB	2.1	2.8	0.75
8070194	RUNX1	1.5	2.0	0.76
8168345	ACRC	1.6	2.1	0.76
7902687	CYR61	2.7	3.5	0.77
8028652	ZFP36/TTP	3.5	4.5	0.78
8104568	UNQ1870	2.0	2.5	0.78
8108370	EGR1	5.6	7.1	0.79
7912347	CASZ1	1.8	2.3	0.79
8147344	PPM2C	2.3	2.9	0.79
8040473	RHOB	2.0	2.5	0.80
7919349	ATG9B/APG9L2	2.0	2.5	0.80
8151816	GEM	2.8	3.5	0.80
8086330	AXUD1	4.3	5.4	0.80
8120335	FAM83B	3.5	4.4	0.81
7997740	MAP1LC3B	1.7	2.0	0.81
8132694	IGFBP1	2.4	2.9	0.82
8024485	GADD45B	2.1	2.5	0.82
8112841	HOMER1	2.1	2.6	0.82
8105267	ITGA2	2.9	3.5	0.83
8154381	C9orf150	1.8	2.1	0.83
8148304	TRIB1	3.0	3.6	0.84
8030128	PPP1R15A	1.8	2.1	0.84
7917875	F3	2.1	2.4	0.84
7927827	MYPN	3.2	3.8	0.84
8129562	CTGF/IGFBP8	2.0	2.4	0.85
8023727	DSEL	2.4	2.8	0.85
7923967	YOD1/DUBA8	1.8	2.1	0.85
7957260	GLIPR1	2.7	3.2	0.86
8084880	HES1	1.7	2.0	0.86
7938390	ADM	2.2	2.5	0.86
7975779	FOS	6.8	7.9	0.86
7912157	ERRFI1/MIG-6	3.0	3.5	0.86
8052669	SERTAD2	1.8	2.0	0.87
8100994	CXCL2	13.6	15.7	0.87
7972055	KCTD12	1.8	2.0	0.87
8103873	snoRNA	2.3	2.7	0.88

8113073	ARRDC3	3.4	3.9	0.88
8179704	IER3	2.3	2.7	0.88
8015387	KRT17	2.7	3.0	0.88
8053668	EIF2AK3	1.9	2.2	0.88
8119088	CDKN1A	2.0	2.3	0.88
7992789	TNFRSF12A	1.8	2.0	0.89
7936242	KIAA1754	1.8	2.0	0.89
7912706	EPHA2	2.3	2.5	0.89
8157216	UGCG	2.3	2.6	0.90
7922610	ABL2/ARG	2.1	2.4	0.90
7958202	CHST11	2.4	2.6	0.91
8005449	KRT17	2.2	2.5	0.91
8095680	IL8	4.3	4.8	0.91
7919743	C1orf138	1.8	2.0	0.91
8178435	IER3	2.4	2.6	0.91
8047161	OBFC2A	5.6	6.1	0.91
8081386	NFKBIZ	3.4	3.7	0.91
7954065	GPRC5A	2.0	2.2	0.91
8055688	RND3	2.4	2.6	0.91
8149264	PPP1R3B	2.1	2.2	0.92
8013042	KRT17	2.3	2.4	0.92
7955589	NR4A1	2.6	2.8	0.92
8005847	TNFAIP1	1.8	2.0	0.92
8112615	ENC1	1.9	2.1	0.92
8122807	AKAP12	1.9	2.1	0.93
7974047	SSTR1	2.0	2.1	0.93
8084206	B3GNT5	1.8	2.0	0.93
8149749	TNFRSF10D	2.1	2.3	0.93
8064766	RNF24	1.9	2.1	0.93
8066513	SDC4	2.0	2.1	0.93
8106403	F2RL1/PAR2	4.4	4.8	0.93
8166469	SAT1	2.3	2.5	0.94
8044301	ANKRD57	1.9	2.1	0.94
7930413	DUSP5	2.2	2.3	0.94
7927631	DKK1	3.3	3.5	0.94
8129677	SGK1	2.7	2.8	0.95
8117106	RNF144B	2.3	2.4	0.95
8021470	PMAIP1/NOXA	2.6	2.7	0.95
8015016	TNS4	2.2	2.4	0.95
8011193	C17orf91	2.1	2.2	0.95
8105040	OSMR	2.5	2.6	0.95
7908409	RGS2	3.1	3.3	0.95
8163896	STOM	2.0	2.1	0.96
8116418	GFPT2	2.9	3.1	0.96
8044080	SLC9A2	1.9	2.0	0.96
8095728	EREG	3.2	3.3	0.96
8088745	FRMD4B/GRSP1	4.8	5.0	0.96
8121277	AIM1	2.2	2.2	0.96
8095744	AREG	9.5	9.9	0.96

7943998	NNMT	3.2	3.3	0.96
8091515	GPR87	3.2	3.3	0.96
7919751	MCL1	2.3	2.3	0.96
8116818	BMP6	1.9	2.0	0.96
8042942	HK2	2.3	2.4	0.97
8094520	PCDH7	2.9	2.9	0.97
8025828	LDLR	1.9	2.0	0.97
7916493	PPAP2B	2.1	2.2	0.97
7986383	IGF1R	2.1	2.2	0.97
7908312	PRG4	2.9	3.0	0.97
8162276	NFIL3	5.0	5.2	0.98
8096116	AGPAT9	2.1	2.1	0.98
7985934	SEMA4B	2.4	2.5	0.98
8026047	JUNB	5.4	5.5	0.98
8109773	WWC1	2.0	2.1	0.98
8023735	TXNDC10	2.2	2.2	0.99
8065071	FLRT3	3.4	3.5	0.99
8041781	EPAS1	2.0	2.0	0.99
7965357	GALNT4	2.0	2.0	0.99
8005785	KSR1	2.1	2.1	0.99
8147112	CA13	3.0	3.0	0.99
7964834	CPM	2.5	2.5	0.99
7982868	CHAC1	2.2	2.2	0.99
8095723	EPGN	3.5	3.5	0.99
8161701	TMEM2	3.6	3.6	1.00
8023688	SERPINB3(4)	18.3	18.4	1.00
8006433	CCL2/MCP1	30.4	30.5	1.00
8148184	FAM83A	2.3	2.3	1.00
7933084	NAMPT	2.2	2.2	1.00
8068593	ETS2	2.5	2.4	1.00
7906400	IFI16	3.3	3.3	1.00
8150592	CEBPD	3.4	3.4	1.01
8111677	LIFR	2.0	2.0	1.01
8018864	SOCS3	6.2	6.1	1.01
7926900	MAP3K8/Tpl-2	2.3	2.3	1.01
8142120	NAMPT	2.2	2.1	1.01
8072710	APOL6	2.9	2.8	1.02
8067113	ZNF217	2.3	2.2	1.02
7967322	GPR109B	2.4	2.4	1.02
8130556	SOD2	2.3	2.2	1.02
8136115	FAM40B	2.4	2.3	1.03
8123598	SERPINB1	2.6	2.5	1.03
8097910	FGB	11.8	11.5	1.03
8077441	BHLHB2	2.4	2.3	1.03
7925062	SIPA1L2	2.1	2.1	1.03
7965040	PHLDA1	2.7	2.7	1.03
7978644	NFKBIA	2.0	2.0	1.04
8069553	NRIP1	2.4	2.3	1.04
8066214	TGM2	2.1	2.0	1.04

8140840	STEAP4	2.3	2.2	1.05
8114010	IRF1	4.4	4.2	1.05
8077851	HRH1	2.9	2.7	1.06
8103311	FGA	8.3	7.8	1.06
8025601	ICAM1	2.9	2.7	1.06
8062461	LBP	2.4	2.2	1.06
7943413	BIRC3	2.2	2.1	1.06
7994280	IL4R	3.2	3.0	1.06
8091327	PLSCR1	2.3	2.1	1.08
7926677	OTUD1/DUBA7	2.2	2.0	1.08
7922976	PTGS2/COX2	5.2	4.8	1.08
8121257	PRDM1	2.5	2.3	1.08
7922717	RGS16	2.6	2.4	1.08
7931353	PTPRE	2.1	2.0	1.09
8122265	TNFAIP3/A20	3.4	3.1	1.09
7957551	SOCS2	2.4	2.2	1.09
8117840	TRIM40/RNF35	3.4	3.1	1.09
7944769	GRAMD1B	3.6	3.3	1.10
7904361	FAM46C	2.7	2.5	1.10
8177750	TRIM40/RNF35	2.9	2.6	1.10
7968236	RASL11A	2.5	2.3	1.10
8019486	SECTM1	3.4	3.0	1.11
8043995	IL1R1	5.6	5.0	1.11
8012949	FBXW10	3.0	2.7	1.12
8023696	SERPINB3	48.9	42.3	1.16
8061564	ID1	3.1	2.6	1.18
8122365	GPR126	2.9	2.5	1.19
8054712	IL1A	2.7	2.2	1.20
7905929	EFNA1	2.6	2.1	1.25
7946983	SAA2	2.9	2.3	1.29
7965335	DUSP6	2.9	2.2	1.33
8103326	FGG	10.5	7.4	1.41
